# Weak-disturbance imaging and characterization of ultra-confined optical near fields

**DOI:** 10.1038/s41377-025-01951-6

**Published:** 2025-10-04

**Authors:** Liu Yang, Yaolong Li, Jinglin Tang, Zhanke Zhou, Hongliang Dang, Zhaohang Xue, Xiaofang Li, Zini Cao, Yijie Luo, Hong Yang, Xiongyong Hu, Wei Wang, Xin Guo, Pan Wang, Guowei Lyu, Qihuang Gong, Limin Tong

**Affiliations:** 1 New Cornerstone Science Laboratory, State Key Laboratory of Extreme Photonics and Instrumentation, College of Optical Science and Engineering, Zhejiang University, Hangzhou, 310027 China; 2 State Key Laboratory for Mesoscopic Physics & Department of Physics, Collaborative Innovation Center of Quantum Matter and Frontiers Science Center for Nano-optoelectronics, Peking University, 100871 Beijing, China; 3 Peking University Yangtze Delta Institute of Optoelectronics, Nantong, 226010 China; 4 Collaborative Innovation Center of Extreme Optics, Shanxi University, Taiyuan, 030006 China; 5 Jiaxing Key laboratory of Photonic Sens-ing & intelligent imaging, intelligent Optics & Photonics Research center, JiaxingResearch institute Zhejiang University, Jiaxing, 314000 China

**Keywords:** Sub-wavelength optics, Nanophotonics and plasmonics

## Abstract

Ultra-confined optical fields are of great importance in fundamental optics and optical technologies. The extreme field confinement in ultra-small nanostructures presents significant challenges in direct near-field characterization. Conventional scanning near-field optical microscopy encounters difficulties in characterizing sub-10-nm confined light fields due to significant disturbances of the optical field caused by the probe. Here, by employing a high spatial-resolved photoemission electron microscopy (PEEM), we succeeded in imaging the ultra-confined near fields of a nanoslit mode in a coupled nanowire pair (CNP) with weak disturbance for the first time and demonstrating a quasi-three-dimensional field distribution of the nanoslit mode. We also show that a PEEM image can identify fabrication defects that are influential to the confined field but are imperceptible to many other means. These results open an opportunity for weak-disturbance characterization of ultra-confined optical near fields, which is an essential step toward future optical devices or technology relying on ultra-confined light.

## Abstract (web summary)

By employing a high spatial-resolved photoemission electron microscopy, we demonstrate near-field imaging and characterization of ultra-confined optical near fields in nanoslits with weak disturbance.

## Graphical abstract

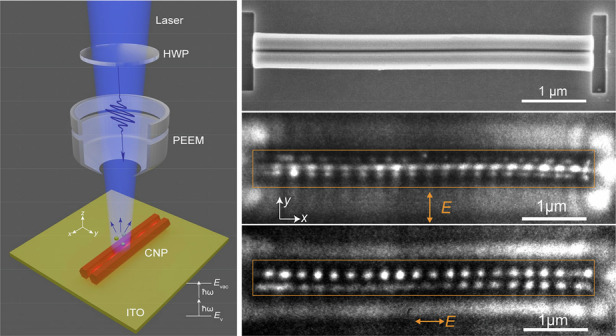

## Introduction

Pursuing an optical field with tighter spatial confinement has always been a fundamentally important issue and an essential step to pushing the limits of a broad range of optical technologies ranging from nonlinear optical processes^1–3^ to super-resolution microscopy^4–6^. In recent years, ultra-confined optical fields have been reported in both metal and dielectric nanostructures, with the field confinement down to sub-10-nm^7–10^ or even sub-1-nm atomic-scale levels^1,5,11–14^. In particular, relying on coherent oscillation of polarized bound electrons around a 1-nm-width slit in a coupled nanowire pair (CNP), a sub-nm-confined optical field has been realized in a nanoslit waveguiding mode supported by the CNP^12–14^. Owing to its dielectric confinement with a diffraction-limited background field, this kind of ultra-confined optical field has low loss and less momentum mismatch during the confining process, and is thus promising for exploring light-matter interactions and related technologies on sub-10-nm or even sub-1-nm level. However, as such a field exists only in the near field of nanostructures with ultra-small feature sizes, its direct imaging which is critical to its characterization and applications, remains challenging.

Typically, scanning near-field optical microscopy (SNOM) can effectively characterize optical near fields with confinement down to the 10-nm level^15–17^. However, to image and characterize a near-field confined tighter than 10 nm, a closer probe-sample distance is required. In that case, the approaching of a typical SNOM probe will significantly influence the original field due to the relatively large probe size and consequently nonnegligible change in the dielectric environment, and thus make the characterization complex and challenging. When the confinement goes down to the sub-nm level, the huge disturbance from a conventional SNOM probe will make the characterization completely ineffective.

Photoemission electron microscopy (PEEM), leveraging the photoelectric effect, has been extensively employed to investigate optical near-field modes, including surface plasmons^18–30^ and dielectric modes^31–34^. By imaging optical-field-excited electrons (photons in, electrons out), PEEM has almost no disturbance to the original optical field as the density of induced photoelectrons^35^ is far lower than that of the polarized electrons. The photoelectrons collected by the imaging system originate from the sample surface, with a mean free path (e.g., a few nanometers for visible light^36^) closing to the near-field decay length of the sub-nm-confined fields^12^. Furthermore, photoelectron emission is a nonlinear process involving multiphoton absorption^37^, with photoemission intensity proportional to |*I* |^*n*^ (where |*I*| is the light field intensity and *n* is the nonlinear order), obviating complex optical field inversion processes. Besides, PEEM’s capability for rapid single-shot projection imaging facilitates convenient near-field characterization of large-area samples. It is worth noting that for techniques with electron beam excitation (e.g., cathodoluminescence (CL)^38^, electron energy loss spectroscopy (EELS)^39,40^, and photon-induced near-field electron microscopy (PINEM)^41,42^), illumination with high-energy electrons may damage dielectric samples. Specifically, the working wavelength of the CL technique is intrinsically limited by the material’s cathodoluminescent spectrum, while EELS and PINEM are typically performed within a transmission electron microscope (TEM), imposing requirements on samples thickness. The photon excitation in PEEM causes less damage to samples than electron excitation, and the parameters of the input laser pulses can be flexibly controlled.

In this work, we utilize high spatial-resolved PEEM to characterize sub-nm-confined nanoslit modes for the first time. We excite the nanoslit mode using vertically polarized incident light, and successfully observe its distinct standing-wave pattern within the mid-gap of the CNP, resulting from the interference between the nanoslit mode and the incident light along the nanowires. The experimentally measured effective wavelength of the nanoslit mode in a ZnO CNP is in good agreement with theoretical calculations. The polarization-dependent evolution of nanoslit modes and near-filed modes in coupled nanowire triplets are also observed. Furthermore, we demonstrate the quasi-three-dimensional characterization of the nanoslit mode, showing that the hotspot is located precisely at the central slit in three-dimensional space. Finally, owing to the high sensitivity of the photoemission signals and fast imaging capabilities of PEEM, we can effectively identify fabrication defects in the CNPs, such as slightly large slit widths and non-uniform nanowires, which are difficult to identify through conventional morphological characterization approaches.

## Results

### Near-field imaging of nanoslit modes

**Fig. 1:**
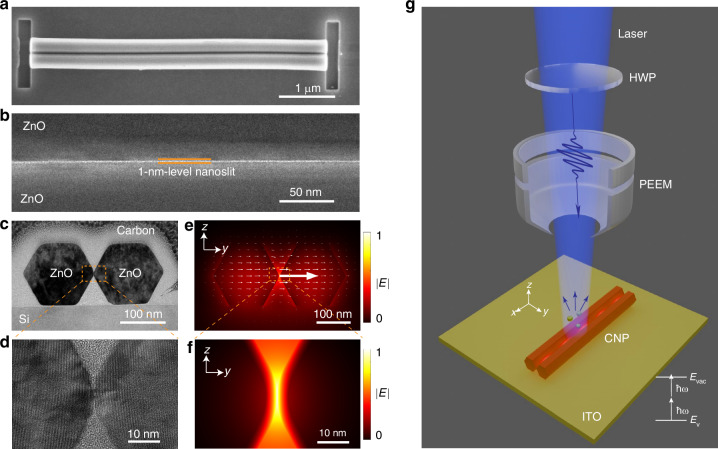
Characterization of ZnO coupled nanowire pairs (CNPs) and photoemission electron microscopy (PEEM) setup scheme. **a** Scanning electron microscopy (SEM) image of a typical ZnO CNP. **b** High-resolution transmission electron microscope (HR-TEM) image of a typical ZnO CNP around the slit. **c** Sectional HR-TEM image of a typical ZnO CNP. **d** Close-up sectional HR-TEM image of the ZnO CNP in (**c**) around the slit. **e** Calculated cross-sectional electric field of TE_0_-like mode with a nanowire diameter of 270 nm and a slit width of 1 nm on a glass substrate with a 20-nm-thickness ITO layer at a 420-nm wavelength. **f** Close-up of the calculated electric field around the slit in (**e**). **g** Schematic of PEEM experimental setup. HWP half-wave plate. *E*_v_, valence band. *E*_vac_, vacuum level. $\hslash$ω, photon energy

In our experiments, wide-bandgap ZnO nanowires (bandgap, ~3.37eV^43^) are synthesized via a bottom-up vapor-liquid-solid method^43^ (see “Materials and methods”), which exhibit atomic-level roughness on their sidewalls (Fig. S1). Using a home-made high-precision micro-manipulation system, we align and assemble two identical nanowires closely along the edges to form a CNP (see “Materials and methods”). The scanning electron microscope (SEM) image of a typical CNP is shown in Fig. 1a, with flat end faces milled by a focused ion beam (FIB). Owing to the sub-nm roughness of the nanowire’s sidewall, an ultra-narrow nanoslit is naturally formed at the center of the CNP. For example, Fig. 1b gives a typical high-resolution transmission electron microscope (HR-TEM) image of such a slit, with a uniform and 1-nm-level slit width. Meanwhile, due to the wurtzite crystal structure, the nanowire has an almost perfect hexagonal cross-section (Fig. 1c), forming a sub-nm slit around the two opposite vertexes (Fig. 1d). By strongly coupling the two lowest-order modes (TE-like and TM-like modes) in each single nanowire, typical CNP waveguides support four lowest eigenmodes (Fig. S6). Among these four modes, owing to coherent oscillation of bound electrons with high charge density around the central slit, the TE_0_-like nanoslit mode (The polarization and wave vector are close to the TE_0_ mode) exhibits a maximum electric field intensity within the slit (Fig. 1e), offering ultra-tight field confinements of ~0.4-nm (*y*-axis) and ~4-nm (*z*-axis) in full width at half-maximum (FWHM) of the electric field intensity (Fig. 1f and Fig. S4). To avoid the accumulation of surface charges during the PEEM experiment, the CNPs are placed on a glass substrate coated with a ~ 20-nm-thickness indium tin oxide (ITO) layer. The introduction of the ultra-thin ITO layer has a negligible influence on optical fields of TE_0_-like modes (Fig. S4), while ensuring high-quality PEEM imaging^34^.

The schematic of the experimental setup of PEEM is depicted in Fig. 1g. A beam of femtosecond pulses, after passing through a half-wave plate for polarization control, is focused on a sample either at normal or at oblique incidence (74^o^ to the normal, see “Materials and methods”). Subsequently, photoelectrons emitted from the sample surface are collected by an electronic lens for imaging. Around a representative operating wavelength of 420 nm, the photoemission (PE) process from ZnO nanowires manifests as a two-photon process to overcome the work function of ZnO (~5.3 eV^44^), which is experimentally confirmed by the power-dependent PE intensity curve (Fig. S3) and is used for mapping the near-field distribution of the nanoslit modes, with a PE intensity proportional to |*E* |^4^, where |*E*| represents the electric field amplitude.

**Fig. 2:**
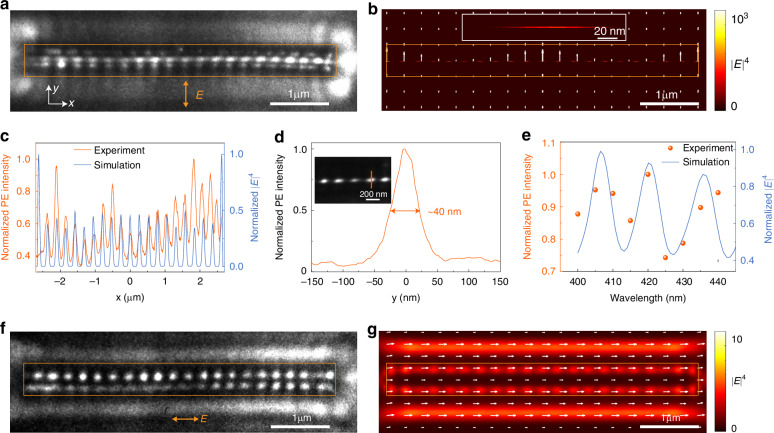
Near-field distribution of nanoslit modes in a CNP. **a** PEEM image of a 5.3-μm-long ZnO CNP with a nanowire diameter of 267 nm under vertical polarization at normal incidence at 420-nm wavelength. The orange rectangular box represents the location of the CNP. **b** Corresponding simulated distribution of |*E* |^4^ in a in the *x*-*y* plane at the position of the slit. The inset represents the close-up distribution of |*E* |^4^ around the slit. The white arrow represents the direction of the electric field. **c** Experimental normalized photoelectrons (PE) intensity and simulated normalized |*E* |^4^ along the *x*-axis at the slit position. The experimental and simulated data are obtained from (**a**, **b**), respectively. **d** Normalized PE intensity along the orange line in the inset. The inset represents the close-up PEEM image of the CNP around the slit. **e** Wavelength-dependent normalized experimental PE intensity and simulated |*E* |^4^. The data are obtained by integrating the PE intensity and |*E* |^4^ within the regions of the orange rectangular box. **f** PEEM image of the ZnO CNP in a with horizontal polarization at normal incidence at 420-nm wavelength. **g** Corresponding simulated distribution of |*E* |^4^ in (**f**) in the *x*-*y* plane at the top surface of the CNP

Experimentally, when exciting ZnO CNPs with a beam of femtosecond pulses with a spot diameter of ~150 μm at normal incidence, incident light couples into the waveguiding modes in the CNP waveguide mainly from the end faces of the CNPs. For a representative ZnO CNP with a nanowire diameter of 273 nm at a wavelength of 420 nm, the CNP waveguide can support four lowest-order strong coupling-induced eigenmodes^12,13^ (Fig. S6). Fortunately, among these four modes, the in-phase-coupling modes (TE_0_-like and TM_0_-like mode, Fig. S6a and Fig. S6b) can be effectively coupled due to the in-phase polarization of the CNP upon excitation by the incident light. Conversely, the coupling channels of π-phase-coupling modes (TE_1_-like and TM_1_-like mode, Fig. S6c and Fig. S6d) are almost blocked. Consequently, the incident light can couple into pure TE_0_-like mode efficiently when the CNP is excited by vertically polarized light (i.e., with the polarization direction perpendicular to the long axis of the nanowire). Due to the interference between the nanoslit mode and the incident light, a characteristic standing-wave pattern emerges at the central slit of the ZnO CNP in a typical PEEM image (Fig. 2a). As a natural Fabry-Pérot (F-P) cavity, the end-face reflectivity of the CNP cavity typically remains low (e.g., ~5% for a CNP with a nanowire diameter of 273 nm at 420-nm wavelength). Therefore, the interference with light reflected from the end face is relatively weak, not observed in the interference pattern. The simulated distribution of |*E* |^4^ in the *x*-*y* plane by Lumerical FDTD (see “Materials and methods”) shows a maximum electric field at the slit with vertical polarization (Fig. 2b). The spatial distributions of experimental PE intensity and simulated |*E* |^4^ at the nanoslit along the *x*-axis are in good agreement (Fig. 2c). The standing-wave pattern satisfies the interference equation^21,26,45^:

1 $$\left({{K}_{{nano}}-K}_{//}\right)* \varLambda =\left(\frac{2{\rm{\pi }}}{{\lambda }_{{neff}}}-\frac{2{\rm{\pi }}}{\lambda }\sin \theta \right)* \varLambda =2{\rm{\pi }}$$

Where *K*_nano_ is the wave vector of the nanoslit mode along the long axis of the nanowire, *K*_//_ is the in-plane wave vector of the incident light, *Λ* is the period of the standing-wave pattern, *λ*_eff_ is the effective wavelength of the nanoslit mode, *λ* is the vacuum wavelength, *θ* is the incidence angle with the normal direction of sample plane. In the case of normal incidence (i.e., *θ* = 0), *Λ* is equal to *λ*_eff_. The *λ*_eff_ obtained from the experimental PE intensity and simulated electric field distribution in Fig. 2c are ~260 nm and ~263 nm, respectively. The consistency of these results confirms that the interference pattern is derived from the near-field nanoslit mode. The slight deviation between experimental and calculated results may be due to the slight deviation in the refractive index of the material and the measurement of topography parameters (e.g., diameter of the nanowire)^46^. When the CNP is excited by an oblique incidence, the period of the standing-wave pattern becomes larger than that in the case of normal incidence (see Fig. S9) as expected. In addition, although the confinement ability of the nanoslit mode is ~1 nm (the inset of Fig. 2b), due to the limit of the spatial resolution of PEEM^18^ and possible surface charge accumulation effect, the measured FWHM (*y*-axis) of the PE intensity is ~40 nm (Fig. 2d). Because the F-P cavity of CNPs modulates the coupling of the nanoslit mode, the wavelength-dependent PE intensity oscillates periodically, which is consistent with the simulation results (Fig. 2e), indicating a quality factor (*Q*) of ~60, and an equivalent lifetime of ~13 fs of the F-P cavity at 406-nm wavelength. At a longer wavelength (e.g., at around 530 nm), the nanoslit mode almost leaks into the substrate, causing a weak mode intensity in the PEEM images (Fig. S10).

Interestingly, when the polarization of the incident light is adjusted to the direction parallel to the nanowire, the position of the standing-wave pattern changes from the central slit to the body of the nanowire (Fig. 2f), due to the coupling of the incident light mainly into the TM-like mode. The extra blurred bright stripes on both sides of the CNP in the image of PEEM (Fig. 2f) can be attributed to the evanescent field of the TM_0_-like mode, which leads to the PE intensity generation from the ITO substrate. The simulated distribution of |*E* |^4^ with the horizontal polarization (Fig. 2g) is in good agreement with the experimental results (Fig. 2f). At the wavelength of 420 nm, the *λ*_eff_ of the TM_0_-like mode is ~248 nm obtained from the distribution of the experimental PE intensity, consistent with the simulated results (~253 nm). Compared with the TE_0_-like nanoslit mode, the maximum of the electric field in the TM_0_-like mode is much smaller (Fig. 2b, g), indicating the ability of the nanoslit mode to enhance the optical field significantly. In addition, we have also measured the total polarization-dependent evolution of the near-field mode, as shown in Fig. S11.

**Fig. 3:**
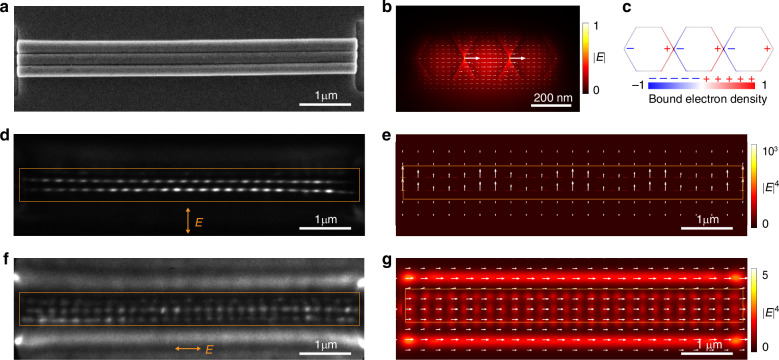
Near-field modes in a coupled nanowire triplet. **a** SEM image of a typical ZnO coupled nanowire triplet. **b** Calculated cross-sectional electric field of the nanoslit mode in a coupled nanowire triplet with a nanowire diameter of 225 nm and a slit width of 1 nm at 395-nm wavelength. **c** Corresponding surface polarized bound charge density distribution of the TE_0_-like nanoslit mode in (**b**). **d** PEEM image of the sample in a with a nanowire diameter of 225 nm under vertical polarization at normal incidence at 395-nm wavelength. The orange rectangular box represents the location of the sample. **e** Corresponding simulated distribution of |*E* |^4^ in (**d**) in the *x*-*y* plane at the position of the slit. **f** PEEM image of the sample in a with horizontal polarization at normal incidence at 395-nm wavelength. **g** Corresponding simulated distribution of |*E* |^4^ in (**f**) in the *x*-*y* plane at the top surface of the nanowire

Similarly, a series of eigenmodes in a coupled nanowire triplet waveguide arise from the strong coupling between eigenmodes in individual nanowires (Fig. S7). More specifically, TE_0_-like mode in the coupled nanowire triplet waveguide originates from the in-phase coupling of the fundamental TE mode in individual nanowires (Fig. 3c), generating two hotspots with strongly confined optical field at the slits (Fig. 3b). The SEM image of a typical coupled nanowire triplet is shown in Fig. 3a. Under a vertically polarized light at normal incident, the TE_0_-like mode in the coupled nanowire triplet is excited, resulting in two-row clear interference patterns at the slits with strong PE intensity (Fig. 3d). While under the excitation of horizontally polarized light, three-row interference patterns appear on nanowire bodies as the incident light couples into the TM_0_-like mode (Fig. 3f). Figure 3e and Fig. 3g plot the simulated distributions of |*E* |^4^ with vertical and horizontal polarizations, consistent with the experimental results. The near-field experimental results of other typical coupled nanowire triplets are shown in Fig. S12.

### Quasi-three-dimensional characterization of nanoslit modes

**Fig. 4:**
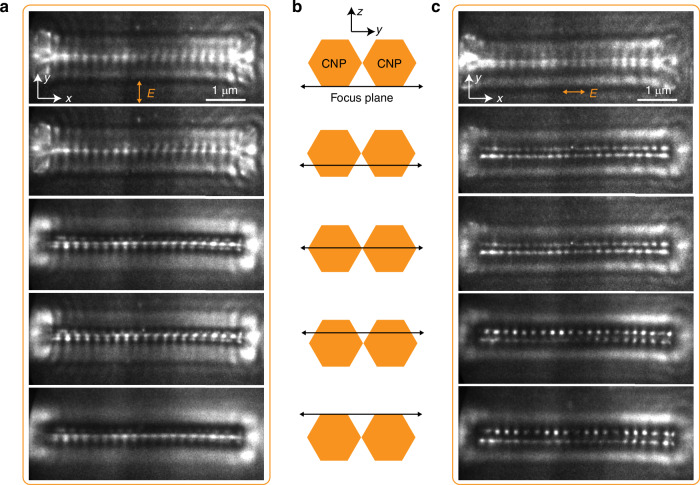
Quasi-three-dimensional characterization of the near-field modes in the CNP. **a** PEEM image of a typical ZnO CNP with vertical polarization at different focal planes. **b** Schematic of the position of the focal plane in the *y*-*z* plane of the CNP. The black arrow represents the focal plane. **c** PEEM image of the ZnO CNP in a with horizontal polarization at different focal planes. All CNPs in Fig. 4 are excited at normal incidence

To further investigate the spatial distribution of the nanoslit modes in the *z*-axis direction, we perform a quasi-three-dimensional characterization of the nanoslit modes by altering the focal plane of the electronic imaging system. Unlike the planar samples typically used in PEEM experiments^30,34^, the CNPs possess non-flat structures with a hexagonal cross-section, while the collected photoelectrons are induced by the electric field on the sample surface. Therefore, it is necessary to image the near-field mode of CNPs along the depth direction (i.e., *z-*axis) in the PEEM experiments. The simulated electric field in the *y*-*z* plane reveals that the location of the electric field maximum is around the slit with vertical polarization, while the hotspots occur on nanowires’ top and bottom surfaces with horizontal polarization (Fig. S8). When focused onto the ITO substrate (bottom surface of CNP), the PEEM images of the CNP modes with vertical and horizontal polarization are both obscure (first rows in Fig. 4a, c). Under the excitation of vertically polarized light, as the focus plane is moved upward, the image of the CNP mode gradually becomes more evident and gets the best imaging contrast around the center height of the CNP (third rows in Fig. 4a), indicating the hotspot is located exactly at the slit (mid-gap of the CNP). By continuing to move the focus plane upward, the PEEM image becomes blurred again (fourth and fifth rows in Fig. 4a). As a comparison, the PEEM images with horizontal polarization become increasingly distinct when moving the focus plane from the bottom to the top surface of the CNP (Fig. 4c). The above results give a quasi-three-dimensional spatial distribution of the nanoslit mode, offering richer evidence for its near-field existence and serving as a demonstration for the three-dimensional reconstruction of the optical fields for bulk samples in PEEM experiments.

### Defect characterization in CNPs

The central electric field of the TE_0_-like nanoslit mode is strongly correlated with the morphology (e.g., slit width and alignment of vertexes) and optical properties of the material around the slit (e.g., refractive index and absorption coefficient). For example, when the slit width increases from 1 to 10 nm, the maximum of |*E* |^4^ of the TE_0_-like mode decreases to 10% of its original value (Fig. S13) due to the diminished oscillation strength of the bound electrons at the interface. Due to the inability to accurately obtain the morphology (Fig. S2) and optical properties, conventional morphological characterization methods (e.g., SEM and TEM) struggle to identify the defects of the CNP samples (e.g., slightly large slit widths and non-uniform nanowires), Therefore, an efficient technique for defects characterization in CNPs is highly desired.

**Fig. 5:**
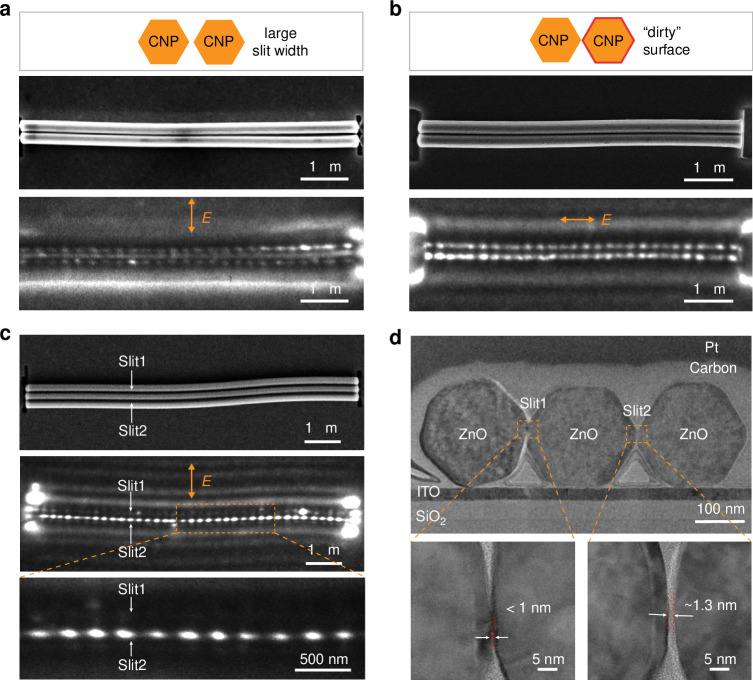
Defect characterization of CNPs in PEEM. SEM (middle row) and PEEM images (bottom row) of CNPs with large slit widths (**a**) and “dirty” surfaces (**b**). The top rows are the schematic of the defects in the CNP. **c** SEM (top row) and PEEM images (middle and bottom rows) of a coupled nanowire triplet. The bottom row is the close-up PEEM image around the slits. **d** Sectional HR-TEM image of the sample in (**c**). The bottom row is a close-up sectional HR-TEM image around the slits

In PEEM characterization, minimal imperfections in the CNP can affect the PE intensity that is proportional to |*E* |^4^. Combined with SEM characterization, we employ PEEM to effectively identify the defects in CNP samples. For example, in the PEEM image of the CNPs with a slightly large slit width (~15 nm), the PE intensity at the slit decreases obviously, while remains an evident contrast against the surrounding background (Fig. 5a), indicating that the central electric field intensity is significantly weakened. Moreover, in addition to local field intensity, the PE intensity is related to the photoemission efficiency of the material’s surface, and is thus a reflection of the nanowire surface uniformity. Although the CNP sample appears almost uniform from the SEM image (middle row in Fig. 5b), the PE intensity at the left end of the CNP is anomalous (bottom row in Fig. 5b), which can be attributed to possible surface contaminations or growth defects on the nanowire.

To verify the ability to characterize optical fields in the CNP sample with more complex morphologies, the PEEM characterization results of a typical coupled nanowire triplet sample are presented here. Despite the difficulty in discerning differences in the morphology of the two slits in the SEM image (top row in Fig. 5c), the PEEM image exhibits a significant contrast in the PE intensity of the two slits (middle and bottom rows in Fig. 5c). The PE pattern of the lower slit (slit2) is distinctly bright, with the PE intensity much higher than that of the upper slit (slit1). The sectional HR-TEM image of the sample is shown in Fig. 5d. Although the width of the slit1 (sub-nm level) is smaller than that of the slit2 (~1.3 nm), the overall tilt of the left nanowire reduces the oscillation strength of the bound electrons at the slit1 interface, while the slit2 exhibits two perfectly aligned opposite vertices with a width of ~1.3 nm. In addition, the slit2 with a larger area has a higher fraction of the total mode power. The above-mentioned technique can also be used for the rapid characterization of minor defects in large-area coupled nanowire arrays with high sensitivity (Fig. S14).

## Discussion

In conclusion, using the weak-disturbance and high spatial-resolved imaging ability of a PEEM, we have successfully demonstrated near-field imaging and characterization of ultra-confined optical near fields in nanoslits for the first time. Compared with many other morphological techniques that are difficult or inadequate for characterizing atomic-scale optical fields, PEEM holds a promising prospect as a powerful characterization tool for extremely confined optical fields with minimal disturbance, such as ultra-confined fields in both dielectric and metal nanostructures^9–11,47^ (Supplementary Note 12). By selecting appropriate material systems (e.g., high photoemission efficiency, balanced conductivity and optical loss) and electron detectors with higher sensitivity (e.g., single-electron level) to reduce the number of photoelectrons involved, the spatial resolution of the PEEM may be further increased to a certain degree (Supplementary Note 12). Also, by depositing atomic-thickness cesium (Cs) to lower the work function^23,25^, the operating wavelengths can be extended from the visible to near-infrared spectrum. Moreover, using a time-resolved PEEM (TR-PEEM) with femtosecond resolution, it is possible to study ultrafast dynamics of an ultra-confined optical near field.

## Materials and methods

### Fabrication of ZnO CNPs

The ZnO nanowires were synthesized via vapor-liquid-solid method^43^ in a high-temperature furnace. A mixture of ZnO powder (Sigma-Aldrich Inc., 99.9% purity) and carbon powder (Sigma-Aldrich Inc., 99.5% purity) with a molar ratio of 1:1 was used as the source placed on an alumina boat in the center of the furnace at a growth temperature of 950 °C. A 300 SCCM nitrogen gas flow transferred the evaporated vapor to silicon wafers in downstream for depositing nanowires. After 200 min under atmospheric pressure, single-crystal ZnO nanowires were synthesized on the Si substrate. Then, nanowires with a suitable diameter were transferred onto a glass substrate coated with an ITO layer. In a homemade high-precision micro-manipulation system based on an optical microscope, the nanowire was cut through a bend-to-fracture process using a fiber taper, ensuring that the nanowires for assembling were almost identical. Finally, after the nanowires were assembled closely to form a CNP using a fiber taper, the end face of the CNP was milled by FIB milling. Before entering the PEEM chamber, CNP samples were annealed under an ultra-high vacuum (~10^–9^ Torr) at 100 °C for 200 min.

### TEM characterization

CNP samples were transferred onto copper grids or SiN_x_ membranes through micro-manipulation for the characterization of nanowire edge roughness and CNP slits in an HR-TEM (The operation voltage is 200 kV). To observe the morphology of slits from a cross-sectional perspective, CNP samples were first deposited with a thick carbon layer and a Pt layer for protection and then thinned to a thickness of ~50 nm along the length direction by FIB milling for HR-TEM imaging.

### PEEM experiments

The PEEM experiments were conducted in a high-resolution, low-energy electron microscopy (LEEM)/PEEM system (ACSPELEEM III, Elmitec GmbH). A commercial Ti: sapphire femtosecond pulse light source (Spectra-Physics Mai Tai HP, pulse duration: 80–100 fs, repetition: 80 MHz) operating at 690–1040 nm was used to pump an optical parametric oscillation (OPO, Inspire Auto 100, Spectra-Physics) to obtain a second harmonic generation (SHG, 390-440 nm) and an OPO signal light (490–750 nm). Another outside BBO was used to produce SHG between 440-500 nm. These lasers were used as the excitation pulses in the PEEM experiment at normal incidence with a spot diameter of ~150 μm or at oblique incidence (74^o^ to the normal) with a spot size of ~30 μm × 70 μm.

### Numerical simulation

In the three-dimensional FDTD simulation, we used the plane wave as a light source and the mesh of the area near slits with a maximum size of 0.2 nm to obtain the distribution of the electric field. In the two-dimensional COMSOL simulation, we calculated the electric field and effective refractive index of the eigenmodes in the CNP structure. The maximum size of the mesh around slits is 0.1 nm. The optical constant of ZnO, ITO, and the glass substrate are obtained from refs. ^48–50^, respectively. For example, the refractive indices of ZnO, ITO and the glass substrate are set to 1.86, 2.00 + 0.008i and 1.53 at 420-nm wavelength in the simulation, respectively.

## Supplementary information

Supplementary Information for Weak-disturbance imaging and characterization of ultra-confined optical near fields

## Data Availability

The data that support the plots within this paper and other findings of this study are available from the corresponding author upon reasonable request.
