# Supplementary material for: Weak-disturbance imaging and characterization of ultra-confined optical near fields
Source: Light Sci Appl. 2025 Oct 4;14:358. doi: 10.1038/s41377-025-01951-6 (PMC12494941; doi:10.1038/s41377-025-01951-6)
Supplement: Supplementary file 1 — Supplementary Information for Weak-disturbance imaging and characterization of ultra-confined optical near fields [file 41377_2025_1951_MOESM1_ESM.docx]

Supplementary Information for

Weak-disturbance imaging and characterization of ultra-confined optical near fields

Liu Yang^†^, Yaolog Li^†^*, Jinglin Tang^†^, Zhanke Zhou^†^, Hongliang Dang, Zhaohang Xue, Xiaofang Li, Zini Cao, Yijie Luo, Hong Yang, Xiongyong Hu, Wei Wang, Xin Guo, Pan Wang, Guowei Lyu*, Qihuang Gong and Limin Tong*

*e-mail: yaolong@pku.edu.cn; guowei.lu@pku.edu.cn; phytong@zju.edu.cn

^†^These authors contributed equally to this work

Supplementary Note 1. Characterization of coupled nanowire pairs

Figure S1 presents a typical high-resolution transmission electron microscopy (HR-TEM) image of a single ZnO nanowire. The sidewall of the whole nanowire is almost uniform (Fig. S1a and Fig. S1b). In a close-up HR-TEM image of the nanowire (Fig. S1c), a distinguishable atomic lattice of single crystal ZnO nanowire can be seen.


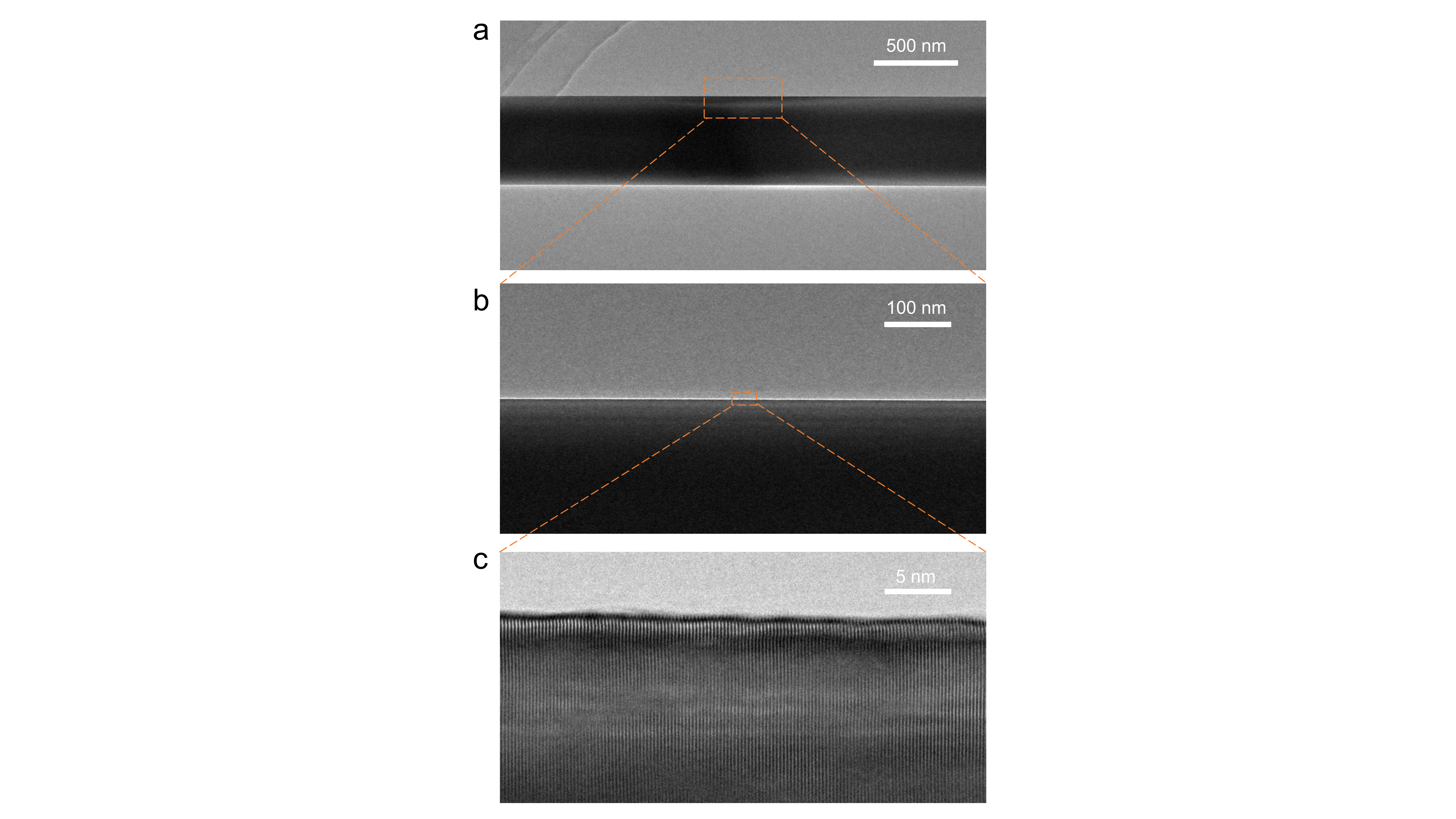


Fig. S1 a HR-TEM image of a typical single ZnO nanowire. b and c Close-up HR-TEM image of the sidewall of the ZnO nanowire in a.

Figure S2 presents SEM and TEM characterizations of a typical ZnO CNP on a SiN_x_ membrane. No additional operations were performed on the sample between the two characterizations. Due to the resolution limitation of SEM (~10 nm for dielectric samples in practice) and the difficulty in assessing the edge of the slit^1^, it is difficult to accurately determine the slit width from the SEM image (Fig. S2a). While TEM can achieve sub-nanometer resolution, it offers only two-dimensional characterization (Fig. S2b), and observing the sample’s cross-section requires destructive thinning (e.g., FIB milling).


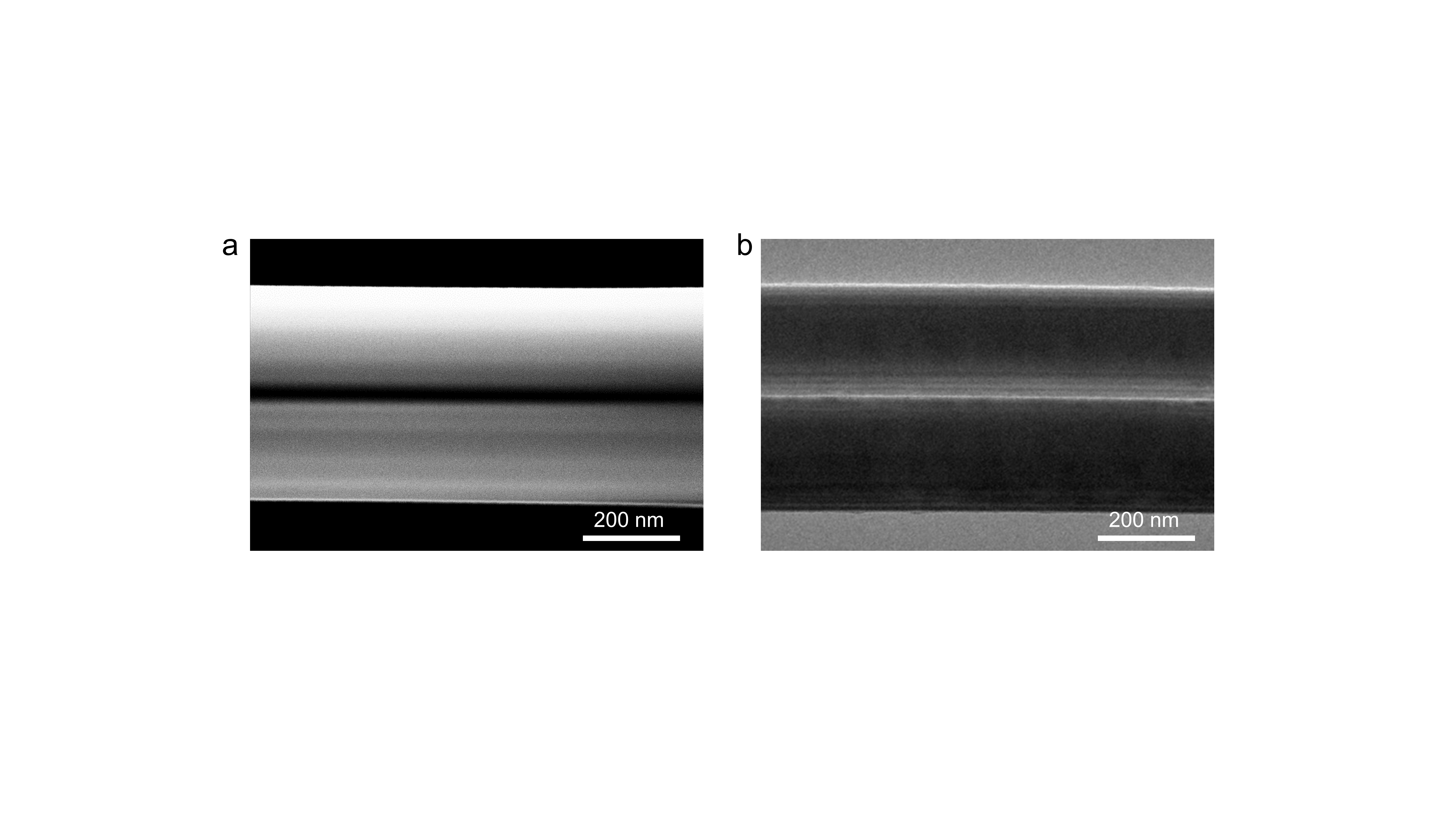


Fig. S2 SEM and TEM characterizations of a ZnO CNP. a SEM image of a typical ZnO CNP. b HR-TEM image of the ZnO CNP in a.

Supplementary Note 2. Power-dependent PE intensity in ZnO CNPs

The PEEM experiment is based on the photoelectric effect. For visible light, such as the typical experimental wavelength of 420 nm, ZnO material absorbs two photons (photon energy, ~2.95 eV @ 420 nm) to overcome the work function (~5.3 eV for typical ZnO nanowire^2^). The processes of multiphoton photoemission are intrinsically governed by the local electric field intensity. The probability of the photoemission process in the two-photon absorption process is proportional to 𝐼^2^ (that is 𝑃 ∝𝐼^2^ ∝|*E*|^4^, where *P* is the PE intensity, *I* is the local field intensity, and |*E*| is the electric field amplitude)^3^. The fitting nonlinear order of power-dependent PE intensity in Fig. S3 confirms the two-photon process. Therefore, for the two-photon process in our experiment, the measured photoelectron distribution is proportional to |*E*|^4^, thus characterizing the ultra-confined optical near fields.


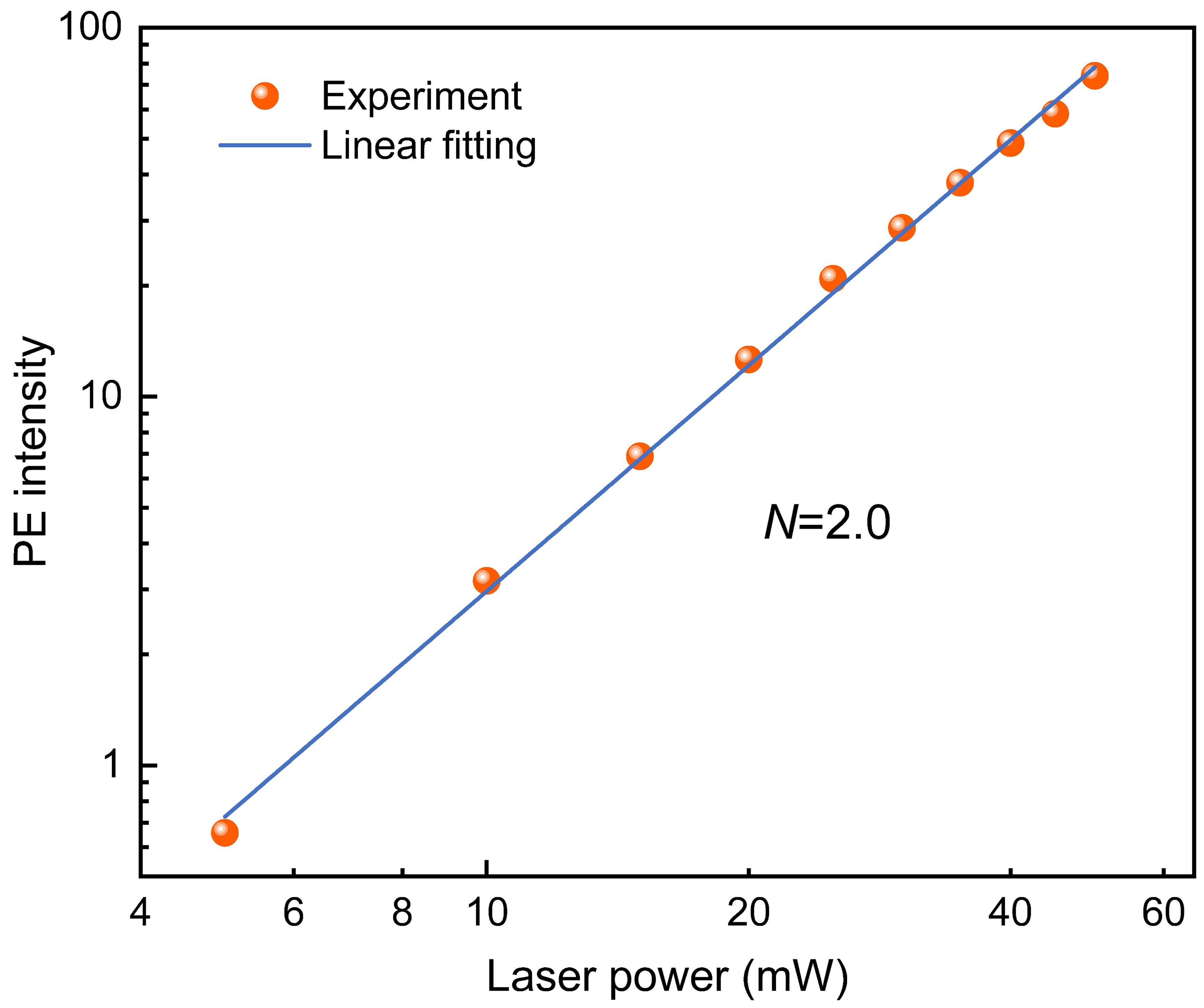


Fig. S3 Power-dependent PE intensity in a typical ZnO CNP. in a typical ZnO CNP with a nanowire diameter of 267 nm under vertical polarization at normal incidence at 420-nm wavelength. The data are obtained by integrating the PE intensity within the region of the CNP sample.

Supplementary Note 3. Influence of the ITO layer and the absorption of the ZnO on nanoslit modes

The electric field distribution and optical confinement of the TE_0_-like nanoslit mode remain almost unchanged with and without the ITO layer (Fig. S4). There are two main reasons. Firstly, the ITO layer is thin enough (~20 nm) and far away from the slit where the hotspot of the TE_0_-like mode locates. Moreover, the polarization direction of the TE_0_-like mode is horizontal which is difficult to couple into the ITO layer^4^.


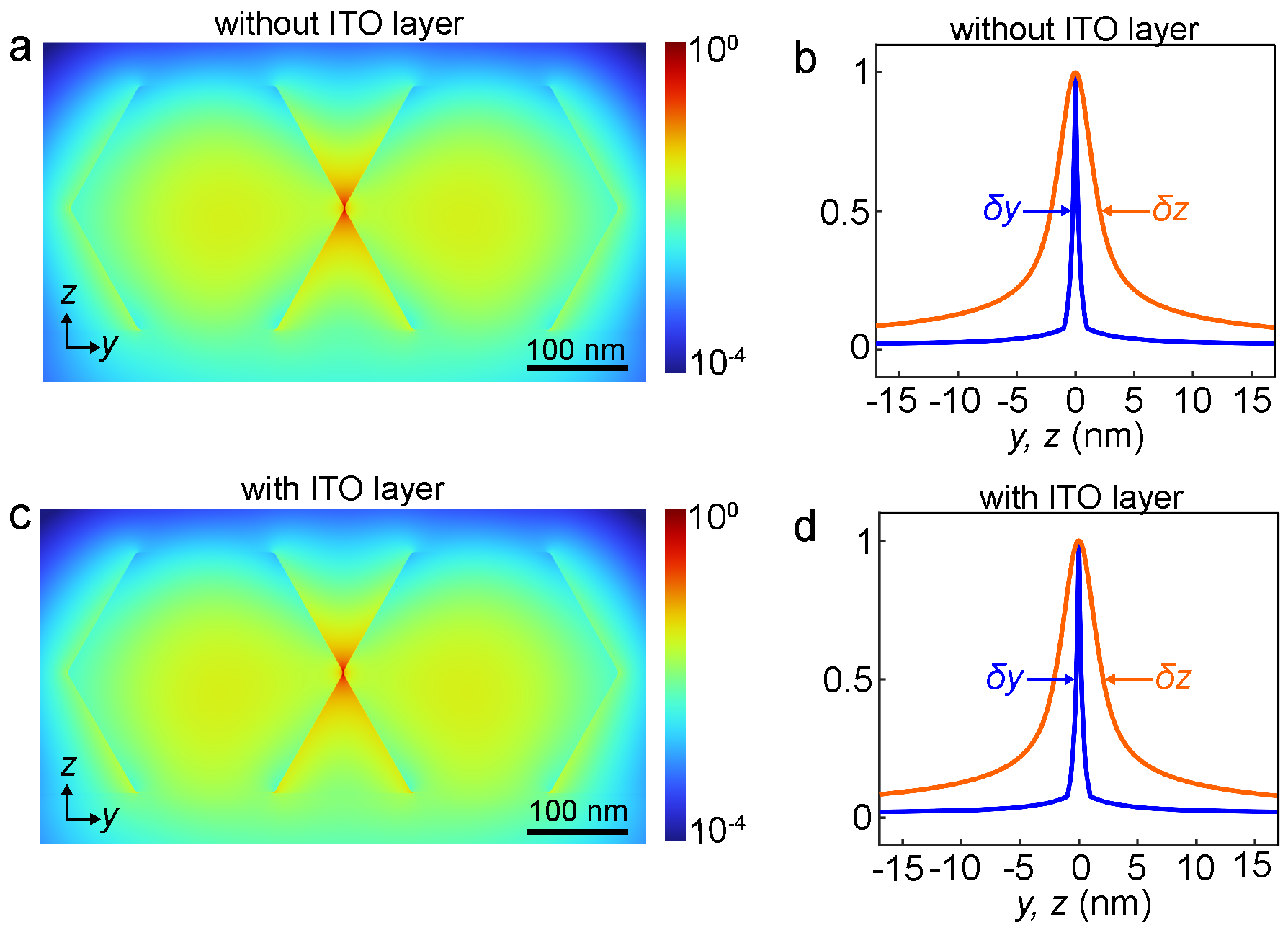


Fig. S4 TE_0_-like nanoslit modes with and without ITO layer. a Normalized cross-sectional field intensity distribution of the TE_0_-like mode of a ZnO CNP with a nanowire diameter of 270 nm and a slit width of 1 nm on a glass substrate without ITO layer. b FWHM of the field intensity along the *y*-axis (blue line) and *z*-axis (orange line) in a. c and d Corresponding results with a 20-nm-thickness ITO layer. The calculated wavelength is 420 nm. The coordinate origin is located at the center of the slit.

To analyze the influence of the absorption of the ZnO material on the TE_0_-like mode, we calculate the TE_0_-like mode in a ZnO CNP waveguide with and without considering the absorption coefficient of the ZnO material, with results shown in Fig. S5. It shows that, the material absorption has negligible influence on the optical field distribution.


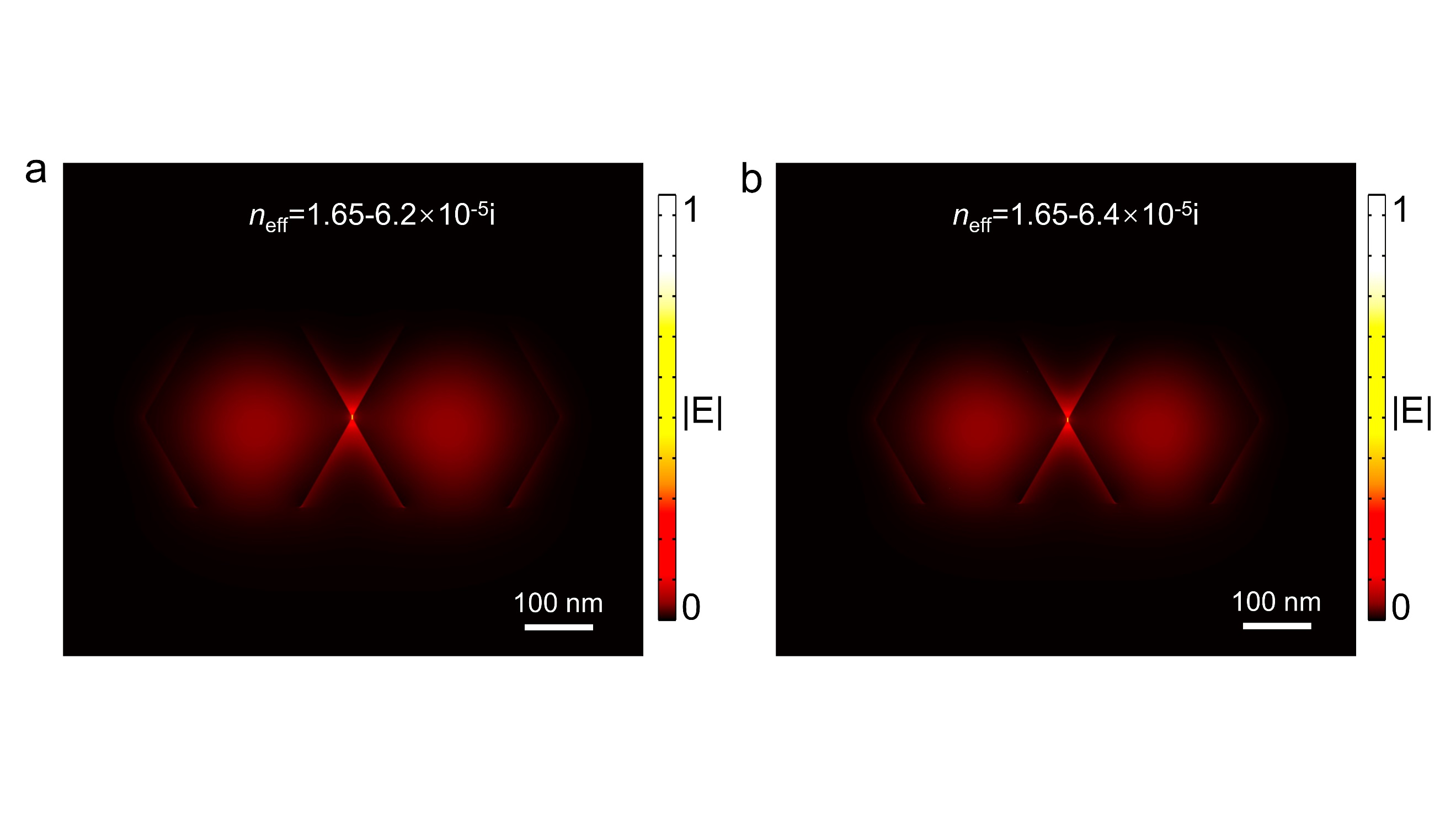


Fig. S5 Normalized electric field distribution of TE_0_-like nanoslit modes in a ZnO CNP with a nanowire diameter of 270 nm and a slit width of 1 nm on a glass substrate with a 20-nm-thickness ITO layer without (a) and with (b) considering absorption coefficient of the ZnO material. The calculated wavelength is 405 nm. *n*_eff_ denotes the effective refractive index of waveguiding modes obtained as the eigenvalue in the mode-solving process.


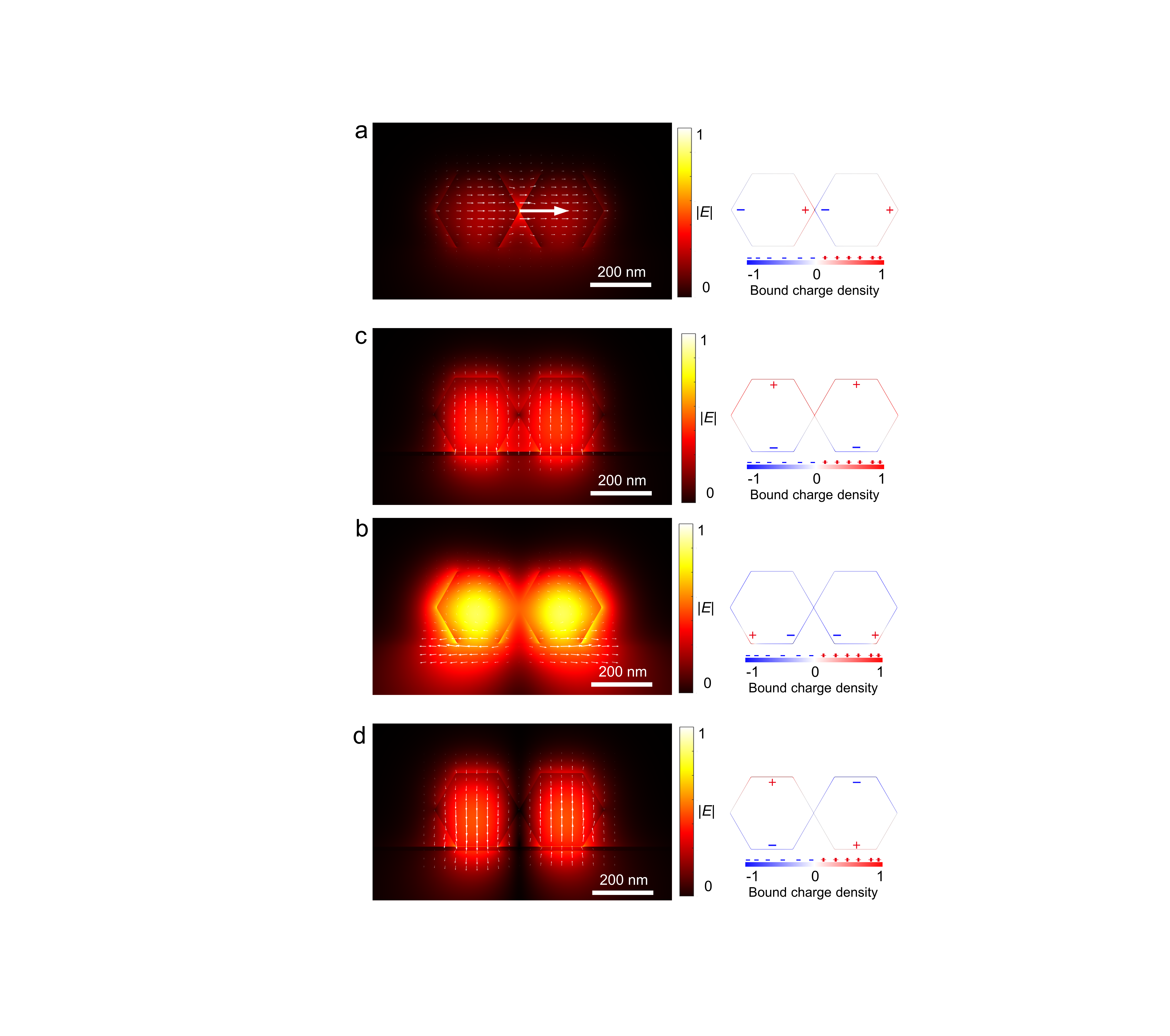


Fig. S6 Four lowest-order eigenmodes in a CNP waveguide. a-d Normalized cross-sectional electric field distribution (left panel) and surface polarized bound charge density distribution (right panel) of the TE_0_-like (in-phase coupling, a), TM_0_-like (in-phase coupling, b), TE_1_-like (π-phase coupling, c), and TM_1_-like modes (π-phase coupling, d) in a ZnO CNP with a diameter of 270 nm at 420-nm wavelength. The TE_1_-like mode in c almost leaks to the substrate due to its effective refractive index being near the glass substrate.

Supplementary Note 4. Eigenmodes in a coupled nanowire triplet


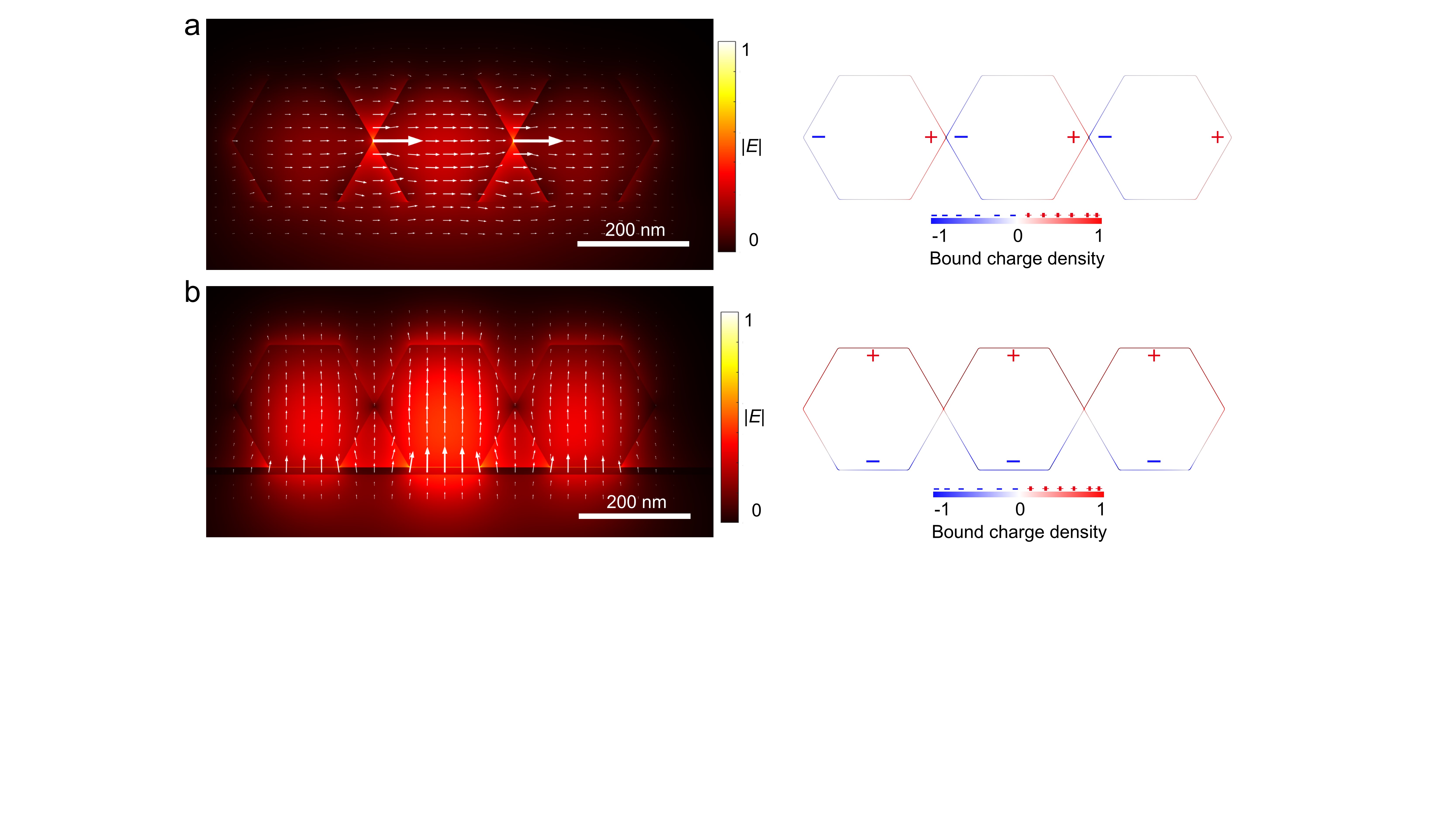


Fig. S7 Eigenmodes in a ZnO coupled nanowire triplet. a and b Normalized cross-sectional field intensity distribution and surface polarized bound charge density distribution of the TE_0_-like (a) and TM_0_-like modes (b) in a ZnO coupled nanowire triplet with a diameter of 225 nm at 395-nm wavelength.

Supplementary Note 5. Distribution of electric intensity in the *y*-*z* plane

Figure S8 plots the distribution of electric intensity in the *y*-*z* plane at normal incident with different polarization. Under the excitation of a vertically polarized light (polarization along the *y*-axis), the maximum value of the electric field is located at the central slit due to incident light couples into the TE_0_-like nanoslit mode (Fig. S8a). Under the excitation of horizontally polarized light (polarization along the *x*-axis), the electric field hotspots on the nanowires' surface are located on the top and bottom surfaces. The photoelectrons induced by the bottom hotspots cannot be collected by the PEEM system. The hotspots inside the nanowire are located at the two outer corners of the central height, which contribute almost nothing during the PEEM imaging process as only the photoelectrons on the surface can be collected by the PEEM system.


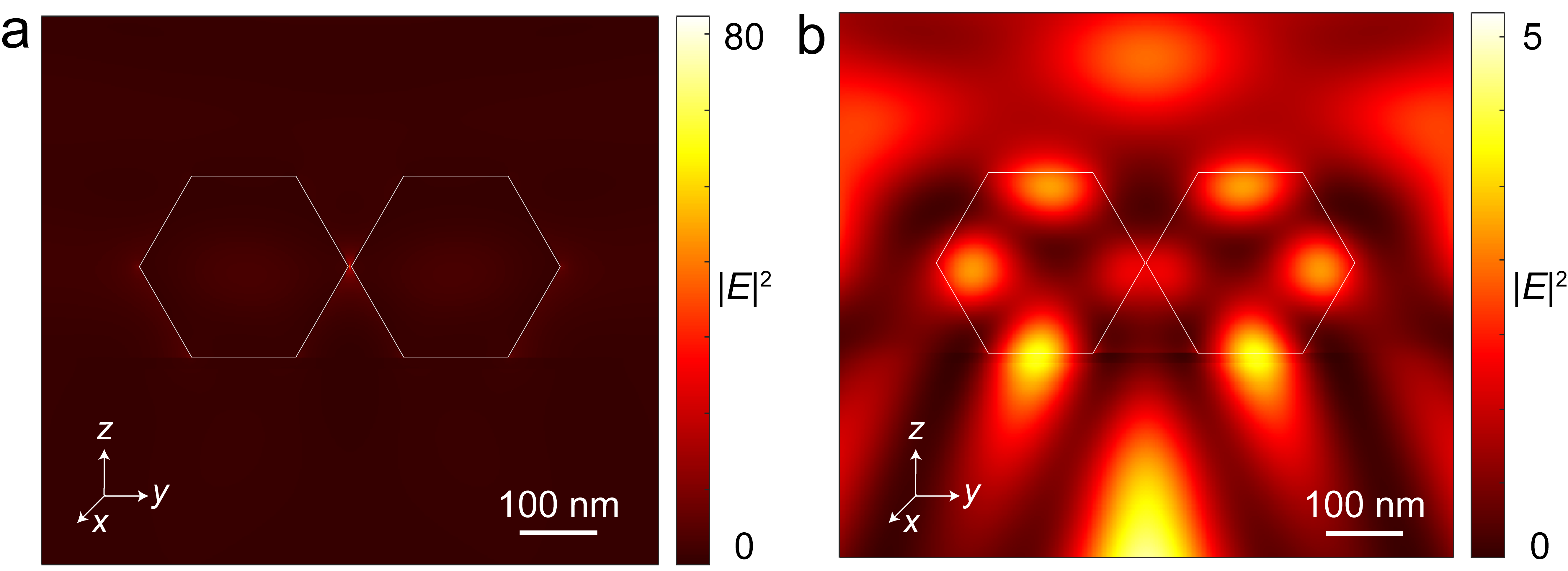


Fig. S8 Distribution of electric intensity in the *y*-*z* plane in FDTD simulation. a and b Distribution of |*E*|^4^ in the *y*-*z* plane of a ZnO CNP with a nanowire diameter of 267 nm with vertical (a) and horizontal polarization (b) at normal incident.

Supplementary Note 6. PEEM images of a ZnO CNP at oblique incident

Figure S9 shows the PEEM images of a ZnO CNP at oblique incident. The period of the interference pattern at oblique incidence is much larger than that of normal incidence, consistent with the interference equation in the manuscript. Meanwhile, standing wave patterns of vertical polarization mainly appear at the slit, while standing wave patterns of horizontal polarization mainly appear on the nanowire body, which is consistent with the cases at normal incidence conditions. The relative ambiguity observed in the interference pattern at oblique incidence may be due to the partial coupling of incident light from the edges of the nanowire in the experiment.


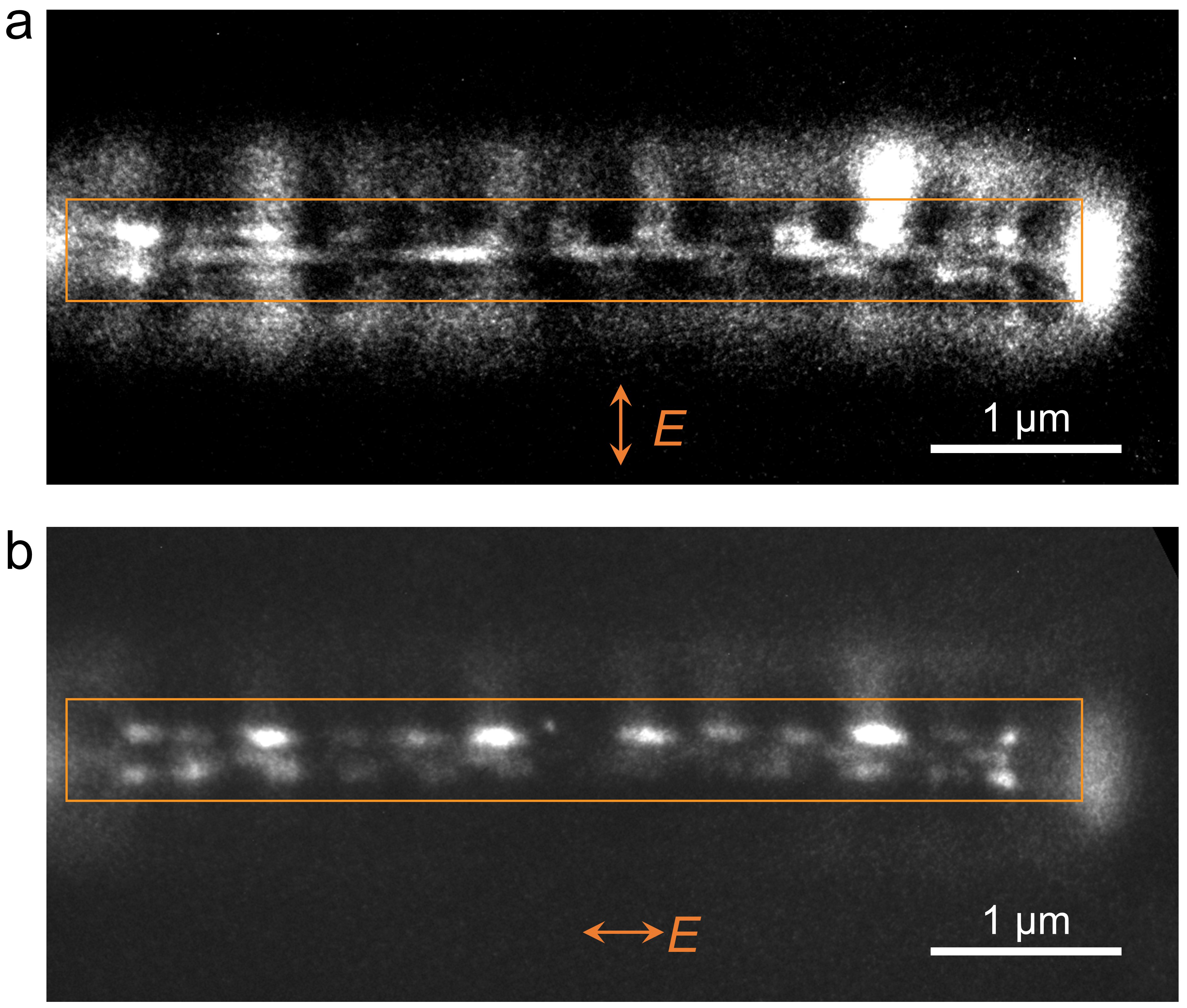


Fig. S9 PEEM images of a ZnO CNP at oblique incident. a and b PEEM images of the ZnO CNP in Fig. 2 of the main text with vertical polarization (a) and horizontal polarization (b) at oblique incident.

Supplementary Note 7. PEEM images of a ZnO CNP at different wavelengths


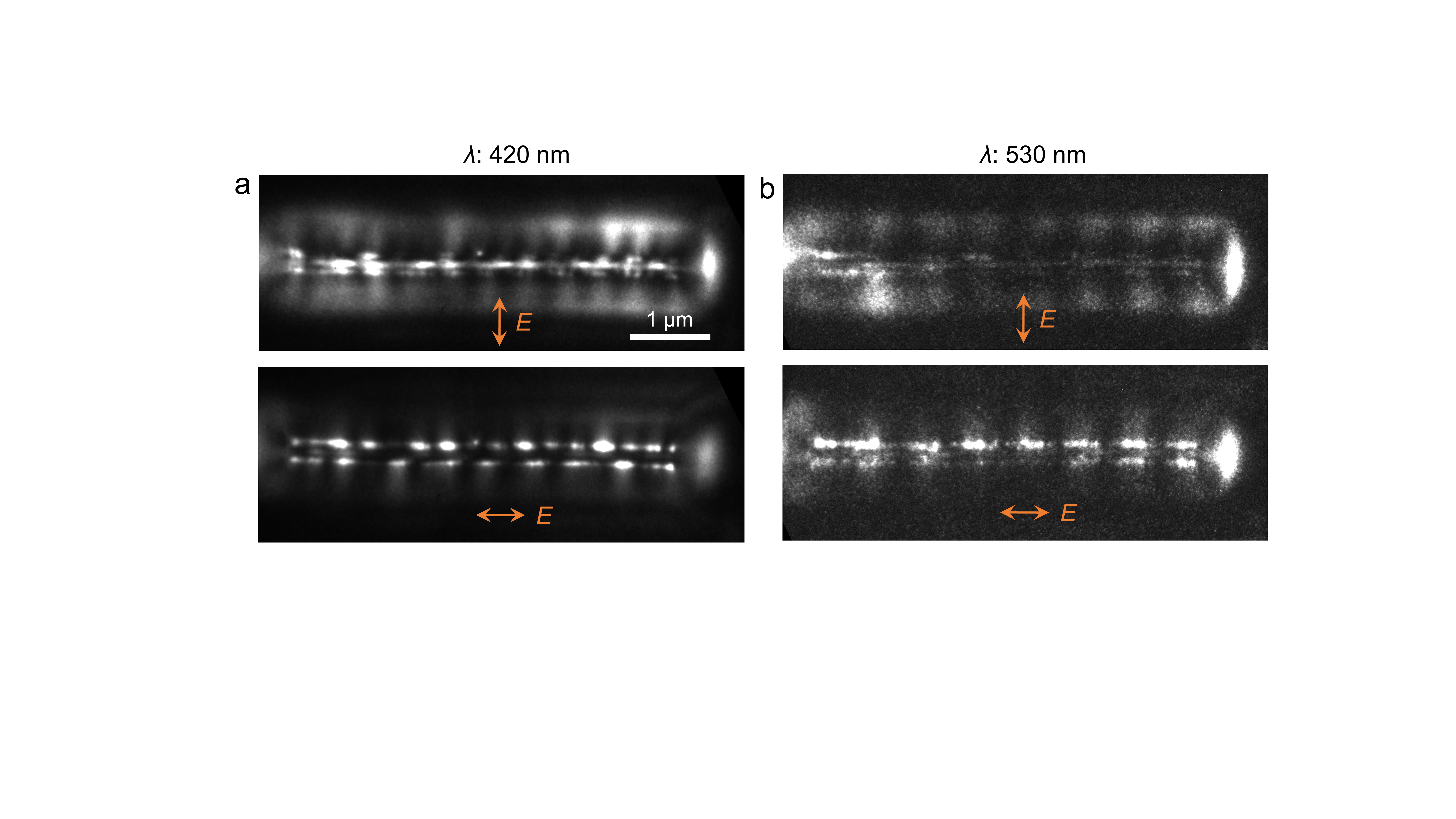


Fig. S10 PEEM images of a ZnO CNP at long wavelengths. a and b PEEM images of a typical ZnO CNP with (top row) vertical and (bottom row) horizontal polarizations at 420-nm (a) and 530-nm (b) wavelengths. The CNP is excited at oblique incidence.

Supplementary Note 8. Polarization evolution of near-field mode in a ZnO CNP


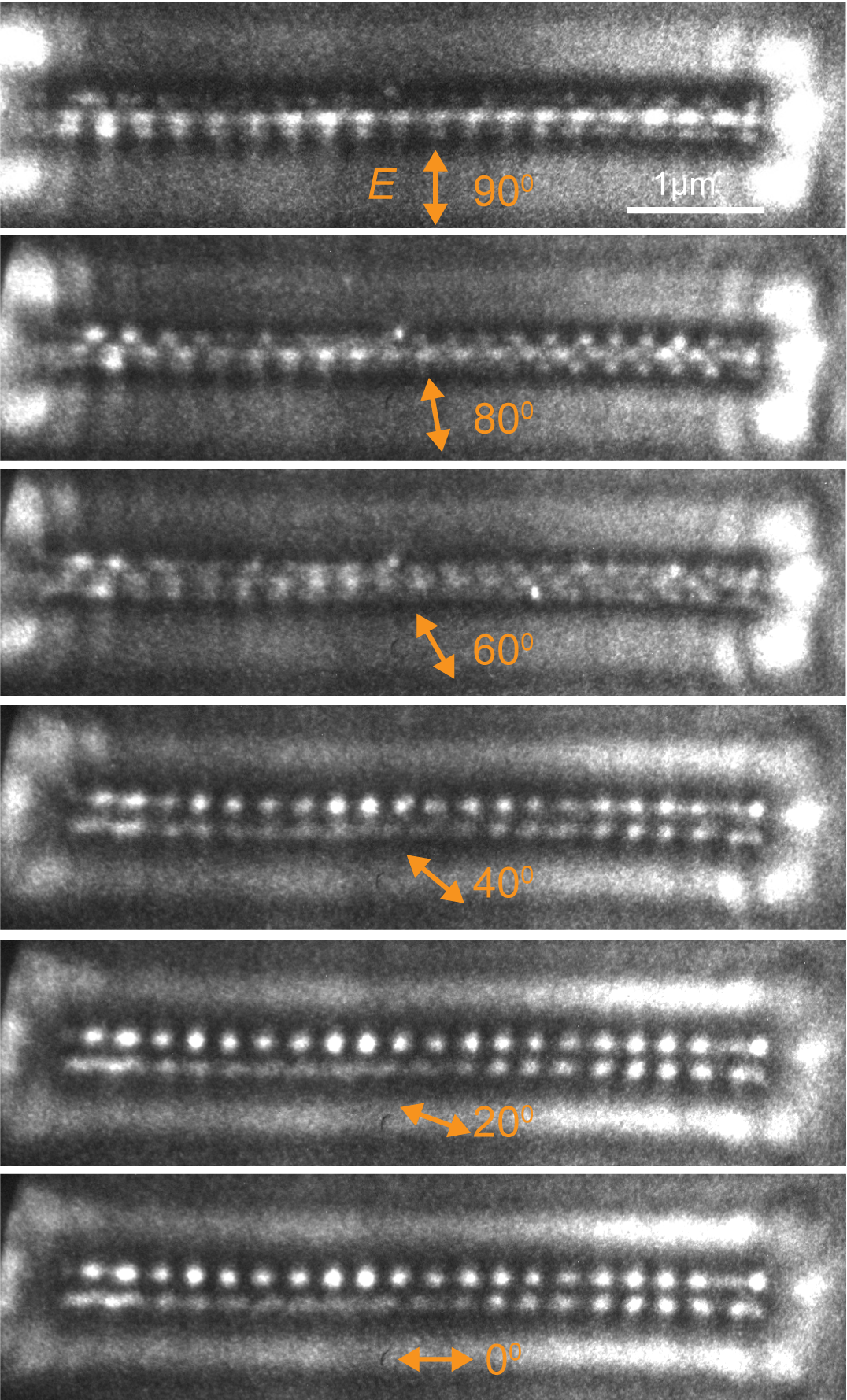


Fig. S11 PEEM images of the ZnO CNP in Fig. 2 of the main text with different polarization at normal incident. The orange arrow represents the polarization direction.

Supplementary Note 9. Near-field distribution of nanoslit modes in a coupled nanowire triplet


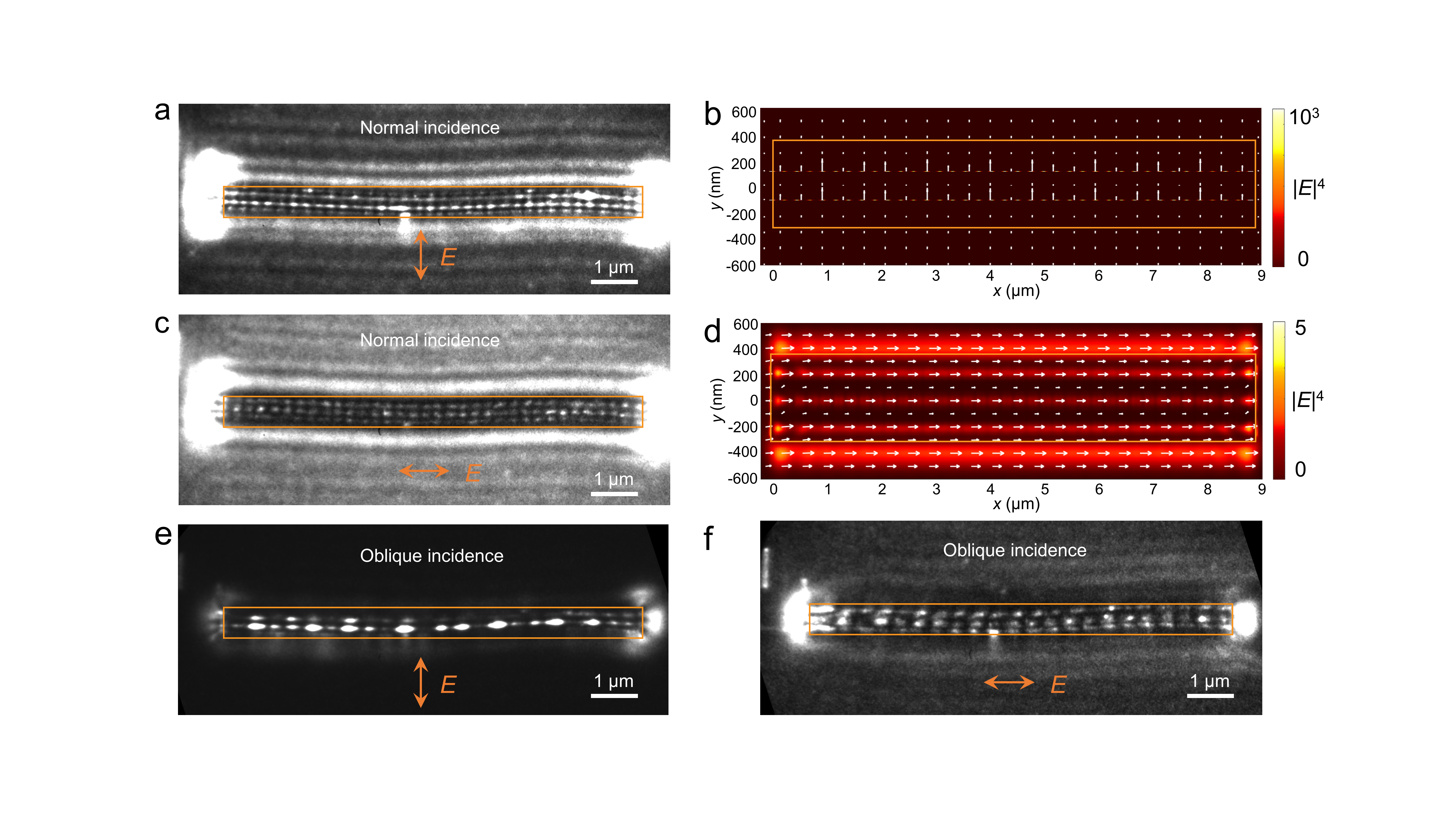


Fig. S12 Near-field modes in a ZnO coupled nanowire triplet. a PEEM image of a typical ZnO coupled nanowire triplet with vertical polarization at normal incidence at 420-nm wavelength. The orange rectangular box represents the location of the CNP. b Corresponding simulated distribution of |*E*|^4^ in a in the *x*-*y* plane at the position of the nanoslit. c PEEM image of the sample in a with horizontal polarization at normal incidence at 420-nm wavelength. d Corresponding simulated distribution of |*E*|^4^ in c in the *x*-*y* plane at the top surface of the nanowire. e and f PEEM images of the sample in a with vertical polarization (e) and horizontal polarization (f) at oblique incidence at 420-nm wavelength.

Supplementary Note 10. Slit-width-dependent maximum of |*E*|^4^ in a ZnO CNP

Figure S13 shows the simulated slit-width-dependent maximum of |*E*|^4^, indicating that the electric field is highly sensitive to the slit width when the slit width is below 10 nm. As shown in Fig. 5c and Fig. 5d, PEEM can recognize 1-nm-level differences in slit width with the help of the ultra-high sensitivity of central fields in the nanoslit mode. As the slit width increases, for example, from ~1 nm to ~15 nm, the ratio of the photoemission intensity at the slit to that of the surrounding background decreases from ~17 to ~3 (Fig. 5a and Fig. 5c). For a larger slit width (e.g., >10 nm), the sensitivity of the PEEM characterization will decrease since the electric field intensity at the slit is weakened, approaching the background field (Fig. S13).

In addition to the slit width, the other morphological parameters of CNP sample, such as vertex curvature and vertex location, also have a significant impact on local optical fields. Consequently, relying solely on morphological characterization makes it quite difficult to accurately simulate the local optical field.


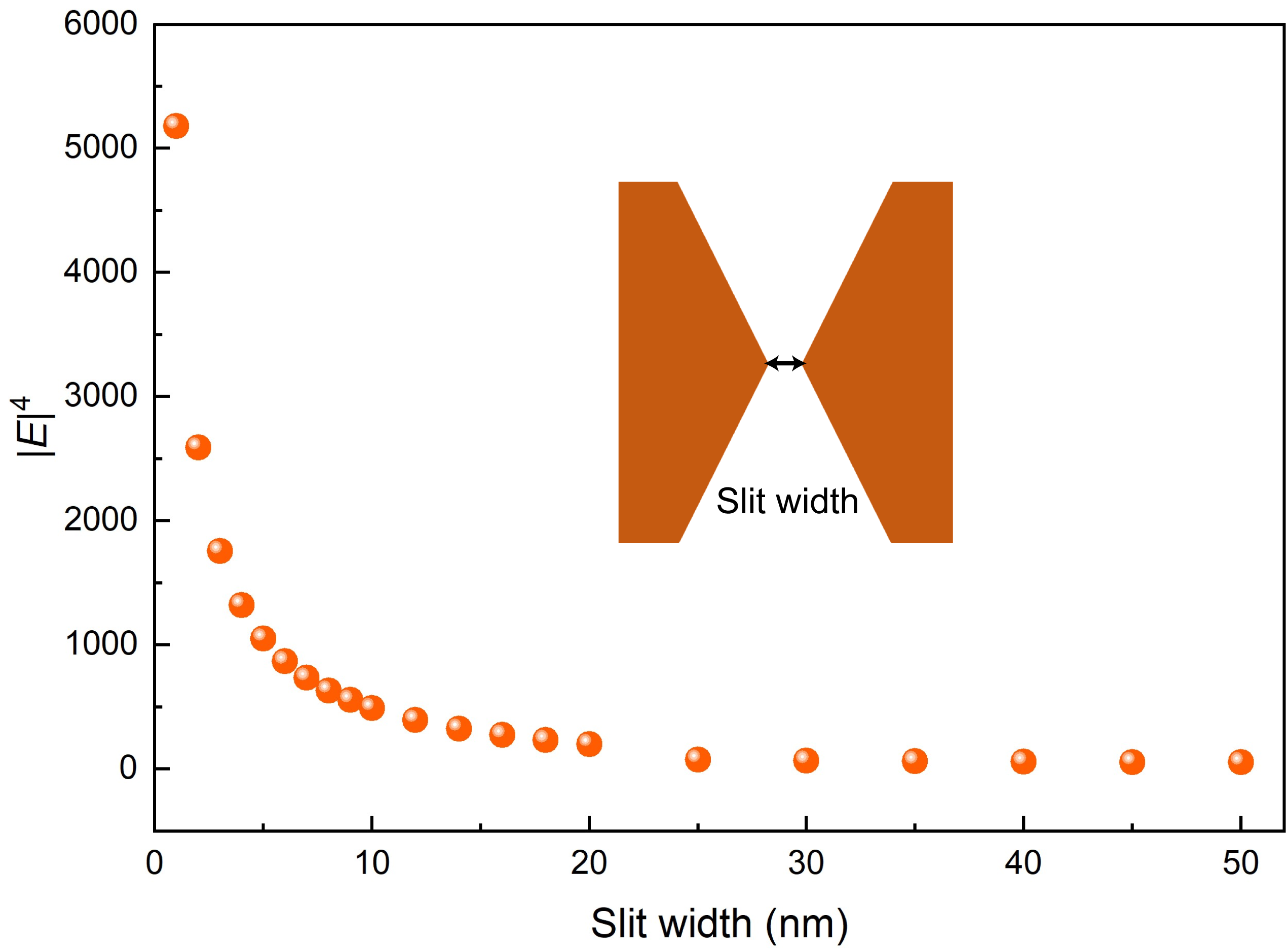


Fig. S13 Slit-width-dependent maximum of |*E*|^4^ in a 267-nm-diameter and 5.3-μm-long ZnO CNP with vertical polarization at 420-nm wavelength. The inset represents the schematic of the slit width in the CNP.

Supplementary Note 11. Defect characterization of multiple-CNPs in PEEM

Owing to the ability of PEEM for rapid single-shot imaging, we demonstrate the near-field detection and characterization of large-area coupled nanowire arrays. In the PEEM image of the array containing five nanowires with vertical polarization (Fig. S14a), the PE intensities at the slits are comparable to that on the nanowire bodies, suggesting imperfect sample preparation. The inhomogeneous PEEM pattern observed in the array containing eight nanowires with horizontal polarization reveals the inherent defect points within the nanowire (Fig. S14b).


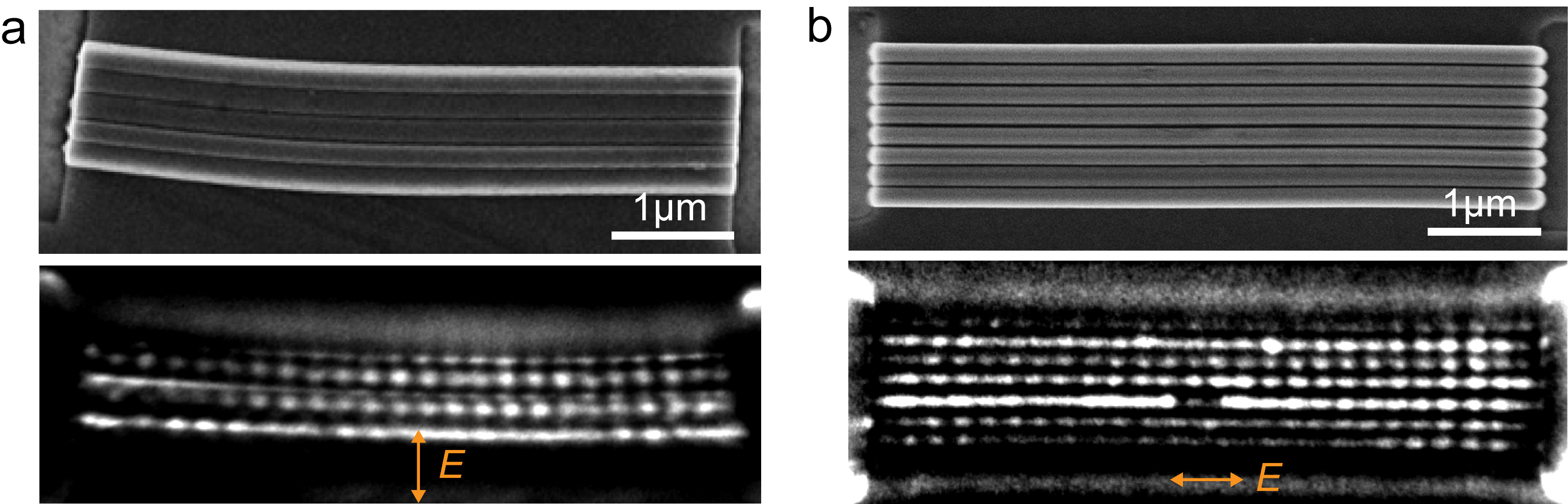


Fig. S14 a-b SEM (top row) and PEEM images (bottom row) of coupled nanowire arrays.

Supplementary Note 12. PEEM for imaging and characterization of ultra-confined optical near fields

Generally, PEEM, as a non-contact and weak-disturbance near-field characterization method, has special advantages in characterizing extremely confined optical fields. For example, through the design of refractive index discontinuity (e.g., typical air-dielectric interface), the bow-tie structures with a nanoscale slit can achieve strong field confinement^5,6^. Due to the hotspot of the optical field being located in the hollow air gap, an SNOM tip will lose feedback during the scanning process in actual experiments, failing in the near-field characterization. The ultra-confined field in a plasmonic nanocavity^7^ can also be characterized by PEEM. PEEM can not only effectively perform optical near-field characterization, but also detect the quality of prepared samples. Unlike SNOM, which has relatively relaxed experimental conditions, PEEM experiments are conducted under ultra-high vacuum, with requirements for conductivity and cleanliness of samples.

Meanwhile, PEEM can characterize various nanomaterials and nanostructures, such as two-dimensional materials^8^, perovskite materials^9^, metal nanostructures^10^, and dielectric nanostructures^11^. Additionally, the view field of PEEM can be large, allowing for large-scale characterization of the sample.

Surface charge accumulation is the main factor affecting the spatial resolution of PEEM characterization. One way to reduce the surface charge accumulation is to improve the conductivity of the sample by material doping. It is possible to use doped ZnO nanowires to reduce the surface charge accumulation effect^12^, without disrupting the optical field confinement below 10 nm. Another way to reduce the surface charge accumulation is to reduce the number of photoelectrons by reducing the intensity of the excitation light to a certain degree, while maintaining a relatively high signal-to-noise ratio in the electron imaging (e.g., using a higher-sensitive electron detector).

References

1 Salvat-Pujol, F. & Villarrubia, J. S. Conventional vs. model-based measurement of patterned line widths from scanning electron microscopy profiles. *Ultramicroscopy* 206, 112819 (2019).

2 Hwang, J. O. et al. Vertical ZnO nanowires/graphene hybrids for transparent and flexible field emission. *J. Mater. Chem.* 21, 3432-3437 (2011).

3 Ferrini, G. et al. Non-linear electron photoemission from metals with ultrashort pulses. *Nucl. Instrum. Methods Phys. Res. Sect. A* 601, 123 (2009).

4 Wu, H. et al. Low-loss photonic-like guided mode in metal-supported optical nanofibers. *Appl. Phys. Lett.* 114, 031104 (2019).

5 Babar, A. N. et al. Self-assembled photonic cavities with atomic-scale confinement. *Nature* 624, 57-63 (2023).

6 Ouyang, Y. H. et al. Singular dielectric nanolaser with atomic-scale field localization. *Nature* 632, 287-293 (2024).

7 Chen, H. et al. Sub-50-ns ultrafast upconversion luminescence of a rare-earth-doped nanoparticle. *Nat. Photon.* 16, 651–657 (2022).

8 Tang, J. L. et al. Direct hot-electron transfer at the Au nanoparticle/monolayer transition-metal dichalcogenide interface observed with ultrahigh spatiotemporal resolution. *Nano Lett.* 24, 2931-2938 (2024).

9 Liu, W. et al. Imaging and controlling photonic modes in perovskite microcavities. *Adv. Mater.* 33, 2100775 (2021).

10 Sun, Q. et al. Direct imaging of the near field and dynamics of surface plasmon resonance on gold nanostructures using photoemission electron microscopy. *Light Sci. Appl.* 2, e118 (2013).

11 Li, Y. L. et al. Revealing low-loss dielectric near-field modes of hexagonal boron nitride by photoemission electron microscopy. *Nat. Commun.* 14, 4837 (2023).

12 Koch, A. et al. Heavily doped zinc oxide with plasma frequencies in the telecommunication wavelength range. *Adv. Photonics Res.* 4, 2200181 (2023).
